# Evaluation of HLA-G and Hematobiochemical levels and their association with malaria and miscarriage among pregnant women: A Ghanaian case-control study

**DOI:** 10.1371/journal.pone.0355847

**Published:** 2026-09-10

**Authors:** Richmond Kwakye, David Courtin, Samuel Asamoah Sakyi, Faustina Pappoe, Gideon Kwesi Nakotey, Naa Adjeley Frempong, Prince Amoah Barnie, Benjamin Amoani

**Affiliations:** 1 Department of medical laboratory sciences, Faculty of health and Allied sciences, KAAF University, Gomoa Fetteh, Ghana; 2 UMR 261 MERIT, Université de Paris, Institut de Recherche pour le Développement (IRD), Paris, France; 3 Department of Molecular Medicine, School of Medical Sciences, Kwame Nkrumah University of Science and Technology, Kumasi, Ghana; 4 Department of Microbiology and Immunology, School of Medical Sciences, University of Cape Coast, Cape Coast, Ghana; 5 Department of Medical Laboratory Technology, Central College of Science and Technology, Agona Swedru, Ghana; 6 Department of Parasitology, Noguchi Memorial Institute for Medical Research, University of Ghana, Accra, Ghana; 7 Department of Forensic Science, School of Biological Sciences, College of Agriculture and Natural Sciences, University of Cape Coast, Cape Coast, Ghana; 8 Department of Biomedical Science, School of Allied Health Sciences, University of Cape Coast, Cape Coast, Ghana; University of Health and Allied Sciences, GHANA

## Abstract

**Background:**

HLA-G (Human Leukocyte Antigen-G) is a non-classical molecule belonging to the major histocompatibility complex (MHC) class I family, located on chromosome 6 within the MHC region, which is pivotal in promoting immune tolerance. The immune-suppressive ability of Human Leukocyte Antigen (HLA-G) and its binding to fetal maternal interface predispose pregnant women to *Plasmodium falciparum* (*Pf*) infection and pregnancy loss, respectively. Alterations in hematological parameters may accelerate the severity of malaria infection. Limited data is available about the effects of malaria infection on hepatocytes and how this endangers the lives of the mother and the fetus due to liver injury. Thus, this study evaluated HLA-G and Hematobiochemical levels and their association with malaria infection and miscarriage among pregnant women in the Ghanaian population.

**Materials and Methods:**

This case-control study recruited forty-six (46) *Pf*-infected pregnant women and forty-six (46) *Pf*-uninfected pregnant women from the Bibiani Anhwiaso Bekwai Municipality, Ghana. A well-structured questionnaire was administered to obtain sociodemographic and clinical data from 21^st^ December 2022–20^th^ December 2023. 10 ml venous blood samples were collected from each participant for *Pf* parasite testing using the *Pf* HRP2 RDT test kit (CareStart TM Malaria PfHRP2/pLDH Ag RDT, Access Bio, Inc., USA), and the results were confirmed with microscopy. Hematobiochemical profile analyses were done using the Sysmex automated hematology and chemistry analyser. Plasma HLA-G levels were quantified using a sandwich ELISA (Maxisop ELISA plate), and their corresponding concentrations were determined.

**Results:**

The results shows a significant difference in the use of IPT-SP and *Pf* infection, with 31(67.4%) for *Pf* positive vs 18(39.1%) for *Pf* negative among those who were not on IPT-SP and 15(32.6%) for *Pf* positive vs 28(60.9%) for *Pf* negative for those who were on IPT-SP (p = 0.007)., Multivariate regression crude analysis shows increased ALT levels among the malaria-infected pregnant women [β = 7.091(95% CI = 1.563–32.182) p = 0.011] compared to the negative group. This finding was insignificant after adjusting for age, IPT-SP use and Trimester. ALP, on the other hand, shows significantly increased levels among the malaria-infected pregnant women compared to the malaria-negative pregnant women for both crude [β = 13.301(95% CI = 1.577–112.208) p = 0.017] and adjusted [β = 17.524(95% CI = 1.534–200.20), p = 0.02] analysis. In a comparative analysis, we found that GLR (G) and PLR (H) levels were significantly higher in participants with *Pf* infections compared to participants without *Pf* infection (*p* < 0.05). We also found that women with a history of miscarriage had considerably higher sHLA-G levels [50.14 (36.30–71.00) vs. 35.44(31.84–42.72)] than women with no history of miscarriage.

**Conclusion:**

*Pf* infection in pregnant Ghanaian women was associated with elevated ALP, GLR, and PLR, indicating hepatic dysfunction and systemic inflammation. The non-use of IPTp-SP among pregnant women was significantly associated with active *Pf* infection, highlighting the importance of chemoprophylaxis. Increased sHLA-G levels in women with miscarriage suggest an immunological link to pregnancy loss. Further prospective investigation of sHLA-G as a biomarker of adverse pregnancy outcome in malaria-endemic settings with a larger sample size is required.

## Introduction

The challenge of malaria persists as a notable threat to global health, particularly within sub-Saharan Africa, where *Plasmodium falciparum* (*Pf*) is recognised as the leading parasitic species [1]. Pregnant women exhibit heightened susceptibility to malaria infection owing to pregnancy-induced immunological alterations and the capacity of *Pf*-infected erythrocytes to sequester within the placental tissue [2,3]. This heightened vulnerability threatens not just the mother's health but also the well-being and growth of the fetus, which may result in serious outcomes like low birth weight, neonatal death, and miscarriage [4].

Malaria infection and pregnancy have complicated interactions that include several immunological and physiological responses. Human Leukocyte Antigen-G (HLA-G), a non-classical major histocompatibility complex (MHC) class I molecule, is an essential part of this complication [5]. During pregnancy, HLA-G is produced from the extravillous trophoblast, which is essential for maintaining immunological tolerance of the mother and fetus [6]. According to studies, HLA-G may also play a role in the immune system's reaction to malaria infection by ensuring infected erythrocyte sequestration, increasing the severity of the illness and pregnancy outcomes [7]. Recurrent miscarriages have been connected to changes in HLA-G expression, which is essential for avoiding immunological rejection of the baby [8]. For instance, a higher incidence of miscarriage has been related to specific HLA-G alleles, such as G*01:04, indicating a genetic predisposition connected with HLA-G variations [9]. While studies have identified associations between specific HLA-G alleles (e.g., G*01:04) and miscarriage risk, the present study focused on circulating soluble HLA-G (sHLA-G) protein levels rather than genetic variants.

The severity of malaria can be monitored using indicators like hematological and biochemical variations [10]. The degree to which hematological and biochemical parameters change is determined by the parasitemia level, nutritional status, immunity to malaria, pregnancy and endemicity of the disease [11]. Hematological abnormalities such as anemia, thrombocytopenia, and variations in white blood cell counts are often seen in malaria-infected pregnant women [12]. The extent of *Pf* infection and its effects on the health of the mother and fetus are also associated with biochemical alterations in liver function indicators such as alanine transferase (ALT), alkaline phosphatase (ALP), and aspartate transaminase (AST) [13].

Although the immune-suppressive ability of HLA-G and its binding at the fetal-maternal interface predispose pregnant women to *Pf* infection and pregnancy loss, studies have reported inconsistent results regarding sHLA-G levels among pregnant women with *Pf* infections. Moreover, there is limited data on the levels of sHLA-G and the hematobiochemical profiles of *Pf*-infected pregnant women within the Ghanaian population. Therefore, this study aimed to evaluate sHLA-G and Hematobiochemical levels and their association with malaria infection and miscarriage among pregnant women in the Bibiani Anhwiaso Bekwai municipality.

## Materials and methods

### Study design and site

This case-control study was conducted at the Bibiani Anhwiaso Bekwai municipality in the Western North region of Ghana. Bibiani Anhwiaso Bekwai municipality is found in the equatorial climatic zone with rainfall averages between 1200 mm and 1500 mm per annum with bimodal rainfall distribution, thus, from March-August and September-October [14,15]. It is a malaria-endemic municipality, making it suitable for the successful implementation of the study.

### Study population

This study recruited 46 *Pf*-infected pregnant women as cases and 46 *Pf*-uninfected pregnant women as controls from the Bibiani Anhwiaso Bekwai Municipality, Ghana, using purposive sampling from 21^st^ December 2022–20^th^ December 2023. The sample size of 46 per group was determined by the availability of eligible participants during the recruitment period and was not based on an a priori power calculation. Participants with a history of prior pregnancy loss were identified through self-report at enrolment, while miscarriage was defined as spontaneous pregnancy loss before 28 weeks of gestation occurring in a previous pregnancy. Pregnant women with chronic complications were, however, excluded from the study.

### Data collection

Sociodemographic and clinical data, except body temperature, were collected from the study participants using a well-structured questionnaire.

### Sample collection

Approximately 10 ml of venous blood was collected into two separate EDTA tubes, each containing 5 ml of blood from each participant. The first EDTA whole blood was used for hematological profiling and malaria screening within two hours of collection. Plasma for HLA-G analysis was separated by centrifugation of the EDTA tube within two hours of collection at 1,500 rpm for 10 minutes using the blood in the second EDTA tube. Plasma aliquots were transferred immediately into labelled cryovials and stored at −80°C until ELISA analysis. Samples were not subjected to repeated freeze–thaw cycles, as single-use aliquots were prepared at the point of separation. Pregnant women who tested positive for HIV, HBV and HCV were contacted for treatment, although excluded from the study.

### Determination of *Pf* infection status

Giemsa-stained thick and thin blood films were read independently by two trained microscopists (certified medical laboratory scientists with at least 5 years of experience in malaria microscopy), blinded to RDT kit (CareStart TM Malaria PfHRP2/pLDH Ag RDT, Access Bio, Inc., USA) results; discordant results between RDT and microscopy, or between the two microscopists, were resolved by repeat testing by a third experienced microscopist. Parasite density was estimated by counting parasites against 200 white blood cells, with density expressed per microlitre using an assumed leucocyte count of 8,000 cells/µL, as described elsewhere [16]

### Determination of hematological parameters

Hematological parameters such as HB, WBC, HGB, and PLT were estimated using an automated hematology analyser (Mindray BC-2800). Whole blood in the K3EDTA test tube was aspirated through the analyser's probe with differential results displayed and printed within 2 minutes. The analyser operated on principles of the impedance method for determining WBC, RBC, and PLT and the colourimetric method for determining HGB [17].

### Determination of biochemical parameters

All biochemical parameters were assayed using serum. The serum was obtained as a result of spinning clotted blood in Serum Separating Test tubes at 4000 rpm for 5 minutes using an 80-2B centrifuge. The serum that was not assayed within 24 hours was stored at 2–8℃ for a maximum of 3 days. Biochemical parameters that were assayed included Total Bilirubin (TB), Aspartate aminotransferase (AST), Alanine aminotransferase (ALT) and Alkaline Phosphatase (ALP). All the biochemical parameters were assayed using a semi-automated chemistry analyser (BC300 VS; 2.6.27.8-Smart-ver 2.0.4) using the Medsource biochemical reagents following the manufacturer's protocol.

### HLA-G quantification

HLA-G concentration in plasma samples was determined using Sandwich Enzyme-linked Immunoassay (ELISA) (Maxisorp ELISA Plate) per the manufacturer’s manual. Briefly, 100μl of capture antibody MEM-G/9 (lot:531294) was pipetted into 10 ml of Phosphate Buffered Saline (PBS) solution in a 15 ml Falcon tube and vortexed for about 1 min to obtain a uniform solution. ELISA microliter plates (MAXISORP) were coated with 100 μL of the solution containing PBS and MEM-G/9 and incubated overnight at 4℃ without agitation. After the overnight incubation, each well of the plate was washed 4 times with 250 μL of a washing buffer (PBS Tween 20: 0.05%) at 1 min intervals.

Additionally, 300μl of blocking buffer was used to block for 1 hour at room temperature with shaking at 400RPM. Each well was again washed 4 times with the washing buffer. Further to this, 50μl of the prepared solution containing 100μl of plasma samples and DAKO diluent, respectively, was transferred into the pre-washed ELISA plate, and 50μl of the DAKO diluent was then added and incubated at room temperature for 2 hours with agitation at 400RPM. Standard HLA-G proteins that were added and diluted serially (two-fold) to estimate concentration in plasma Standards, positive and negative control, blanks and samples were transferred in duplicates. Each well was again washed 4 times using 250μl of the washing buffer with 1 min interval after incubation. Also, 100μl of a secondary antibody solution containing 10 ml of PBS buffer and 1μl of β_2_ microglobulin was transferred into each well of the pre-washed ELISA plate and incubated at room temperature for 1 hour with shaking at 400RPM. Wells were again washed 4 times with 250μl of washing buffer at 1 min intervals. Then, 100μl of an enhancer containing 10 ml of PBS and 50 μl of DAKO Envision System HRP (lot: 10151788: ref: k4003) was added to each well and incubated for 1 hour with shaking at 400RPM. Each well was again washed 4 times with the buffer. Again, 150μl of the substrate Tetramethylbenzidine (TMB) was transferred into each well and incubated at ambient temperature in the dark for 35 minutes without agitation. OD was then measured at 620nm, and 100μl of HCL (IN) was transferred into each well to stop the enzymatic reaction. OD was again measured at 450nm. ODs were converted into arbitrary units using the ADAMSEL software.

### Data management and statistical analysis

Prior to analysis, data were screened for completeness, outliers, and normality. Continuous variables were assessed for normal distribution using the Shapiro–Wilk test and visual inspection of histograms and Q–Q plots. Descriptive statistics were used to summarise participant characteristics. Continuous variables were presented as mean ± standard deviation (SD) for normally distributed data, while categorical variables were expressed as frequencies and percentages. Comparisons between Plasmodium falciparum (*Pf*)-infected and uninfected pregnant women were performed as follows: the independent samples t-test was used for normally distributed continuous variables, while the Mann–Whitney U test was applied for non-normally distributed variables. Chi-square (χ²) test or Fisher’s exact test (where expected cell counts were <5) was used to compare categorical variables. To assess differences in biochemical parameters (e.g., ALT and ALP) between groups, linear regression analysis was employed. Both crude (unadjusted) and adjusted models were fitted, with adjustment for potential confounders, including age, IPT-SP use and trimester. The association between hematological indices (e.g., granulocyte–lymphocyte ratio [GLR], platelet–lymphocyte ratio [PLR], lymphocyte–monocyte ratio [LMR], and ALT levels) and *Pf* infection was evaluated using binary logistic regression analysis. Variables with p < 0.20 in univariate analysis or those deemed clinically relevant were included in the multivariable models to control for confounding. Comparative analyses of HLA-G levels and hematological indices between malaria-infected and uninfected groups, as well as between participants with and without a history of miscarriage, were conducted using appropriate parametric or non-parametric tests based on data distribution. A p-value < 0.05 was considered statistically significant.

### Ethical consideration

Ethical approval was obtained from the University of Cape Coast (UCC) Institutional Review Board (UCCIRB/CHAS/2022/153). Internal approval was also obtained from the management of the hospitals before data collection. Written and signed informed consent was obtained from the study participants and appended signatories to ensure voluntary participation after a candid explanation of the research protocol. All participants who could not read or write were guided by a witness and endorsed by thumb printing.

## Results

### Sociodemographic and clinical characteristics of the study participants

The mean ages obtained for this study were similar among both groups (26.13 ± 5.54 vs 27.09 ± 6.47, p = 0.448) for *Pf-*positive and negative, respectively. Pregnant women in their third trimester recorded the highest number among both groups, although the percentage was higher among the negative (63%) than the positive group (41.3%). In terms of education, junior high level was recorded as the most common among the participants in both groups [21 (45.7%) for *Pf* negative vs 15 (32.6) for *Pf* positive]. Again, the Majority 41 (91.1%) for *Pf* negative vs *Pf* positive 39 (84.8%) among both groups were engaged in informal occupation (p = 0.35). Among the infected participants, 32 (69.9%) did not experience miscarriage, whereas 14 (30.4%) did. In contrast, among the uninfected participants, 17(37%) experienced miscarriage while 29(63%) did not. Surprisingly, each of the participants in both groups who had records of miscarriage had experienced miscarriage two or more times, 2 (11.8%) for *Pf* negative vs 3 (21.4%) for *Pf* positive. The results also saw a significant difference in the use of IPT-SP and *Pf* infection, with 31(67.4%) for *Pf* positive vs 18(39.1%) for *Pf* negative among those who were not on IPT-SP and 15(32.6%) for *Pf* positive vs 28(60.9%) for *Pf* negative for those who were on IPT-SP (p = 0.007). Also, it was observed that participants among both groups frequently used insecticide-treated nets [20 (64.5%) for *Pf* negative vs 19 (63.3%) for *Pf* positive. The mean body temperature recorded among the study malaria negative and positive groups was similar (p = 0.248) (Table 1).

**Table 1:** Sociodemographic and clinical characteristics of the study participants.

Variable	*Pf* Negative (n = 46)	*Pf* Positive (n = 46)	*p*-value
**Age (Years) (π ± SD)**	27.09 ± 6.47	26.13 ± 5.54	0.448^a^
			
**Trimester**			0.105^b^
First	6 (13.0)	8 (17.4)	
Second	11 (23.9)	19 (41.3)	
Third	29 (63.0)	19 (41.3)	
**Educational Level**			0.710^b^
No formal education	7 (15.2)	9 (19.6)	
Primary	7 (15.2)	7 (15.2)	
Junior High	21 (45.7)	15 (32.6)	
Secondary	7 (15.2)	11 (23.9)	
Tertiary	4 (8.7)	4 (8.7)	
**Occupation**			0.354^b^
Informal	41 (91.1)	39 (84.8)	
Formal	4 (8.9)	7 (15.2)	
**Ethnicity**			0.392^b^
Asante	8 (17.4)	6 (13.0)	
Northerner	13 (28.3)	17 (37.0)	
Sefwi	17 (37.0)	11 (23.9)	
Wassa	6 (13.0)	6 (13.0)	
Fante	2 (4.3)	6 (13.0)	
**Previous Pregnancy**	13 (28.3)	13 (28.3)	> 0.999^b^
**Previous Malaria Infection**	15 (32.6)	11 (23.9)	0.559^b^
**Miscarriage**			0.508^b^
No	29 (63.0)	32 (69.6)	
Yes	17 (37.0)	14 (30.4)	
**Frequency of Miscarriage**			0.467^b^
Once	15 (88.2)	11 (78.6)	
Twice or more	2 (11.8)	3 (21.4)	
**IPT-SP**			**0.007** ^b^
No	18 (39.1)	31 (67.4)	
Yes	28 (60.9)	15 (32.6)	
**IPT-SP Frequency**			0.349^b^
Once	4 (14.8)	4 (26.7)	
Twice or more	23 (85.2)	11 (73.3)	
**ITNs Usage**	28 (60.9)	30 (65.2)	0.666^b^
**Frequency of ITNs Usage**			0.923^b^
Sometimes	11 (35.5)	11 (36.7)	
Always	20 (64.5)	19 (63.3)	
**Temperature (π ± SD)**	36.41 ± 0.47	36.56 ± 0.74	0.248^a^

Note: ^a^*P*-values computed by Independent Sample T-test, ^b^ *p*-values computed by Chi-square test, *p*-values < 0.05 and bolded means statistically significant, π- mean

Abbreviations: *Pf*: *Plasmodium falciparum*, IPT-SP: sulfadoxine-pyrimethamine intermittent preventative treatment, ITN: Insecticide Treated Net

### Biochemical levels among pregnant women with or without malaria infection

Multivariate regression crude analysis shows increased ALT levels among the malaria-infected pregnant women [β = 7.091(95% CI = 1.563–32.182) p = 0.011] compared to the negative group. This finding was insignificant after adjusting for age, IPT-SP use and Trimester. ALP, on the other hand, shows significantly increased levels among the malaria-infected pregnant women compared to the malaria-negative pregnant women for both crude [β = 13.301(95% CI = 1.577–112.208) p = 0.017] and adjusted [β = 17.524(95% CI = 1.534–200.20), p = 0.02] analysis (Table 2).

**Table 2:** Biochemical levels among the pregnant women with or without malaria infection.

Variables	Crude ^a^ (Model 1)		Adjusted^b^ (Model 2)	
	β (95% CI)	p-value	β (95% CI)	p-value
TB	0.312(0.94-1.032)	0.056	0.287(0.67-1.234)	0.09
AST	2.455(0.967-6.232)	0.059	1.739(0.627-4.820)	0.28
**ALT**	**7.091(1.563-32.182)**	**0.011**	5.248(0.95-28.8)	0.056
**ALP**	**13.301(1.577-112.208)**	**0.017**	**17.524(1.534-200.20)**	**0.02**

Multivariate regression analysis with and without adjustment estimated the effect of covariates on HLA-G and Biochemical profile.CI: confidence interval. The model (^a^) crude analysis without adjustment and (^b^) adjusted for Age, IPT-SP use, and Trimester. The negative control (pregnant women without *Pf* infection) was set as reference for the model. TB-Total Bilirubin, AST-Aspartate aminotransferase, ALT-Alanine aminotransferase, ALP-Alkaline phosphatase.

### HLA-G and Hematobiochemical Predictors of *Pf* infection among pregnant women

In a univariate logistic regression model, increasing GLR levels (cOR: 1.57, 95% CI: 1.08–2.29; *p* = 0.018) were significantly associated with a 2-fold increased likelihood of *Pf* infection among pregnant women. Also, increasing ALT levels (cOR: 0.94, 95% CI: 0.89–0.99; *p* = 0.009) was significantly associated with a 6.0% decreased likelihood of *Pf* infection among pregnant women.

On the contrary, none of these parameters showed significance for the adjusted odds ratio after adjusting for covariates. *Pf* (Table 3).

**Table 3:** HLA-G and Hematobiochemical Predictors of *Pf* infection among pregnant women.

Variables	cOR (95% CI)	*p*-value	aOR (95% CI)	*p*-value
HLA-G (ng/mL)	1.00 (0.99-1.02)	0.599	1.00 (0.99-1.02)	0.660
LMR	0.91 (0.71-1.16)	0.428	1.07 (0.80-1.43)	0.650
GLR	1.57 (1.08-2.29)	**0.018**	1.49 (0.98-2.29)	0.064
PLR	1.01 (0.99-1.02)	0.128	1.01 (0.99-1.01)	0.306
TB (μmol/L)	1.05 (0.99-1.10)	0.062	1.04 (0.99-1.10)	0.143
AST (U/L)	0.99 (0.97-1.00)	0.072	0.99 (0.97-1.01)	0.323
ALT (U/L)	0.94 (0.89-0.98)	**0.009**	0.95 (0.90-1.01)	0.084
ALP (U/L)	1.00 (0.99-1.00)	0.916	1.00 (0.99-1.00)	0.778

Note: *p*-values computed by logistics regression prediction model, *p*-values < 0.05 and bolded means statistically significant. adjusted for Age, IPT-SP use, Trimester

**Abbreviations:** cOR: Crude odds ratio, aOR: Adjusted odds ratio, CI: Confidence interval, HLA-G: Human Leukocyte Antigen-G, LMR: Lymphocyte-monocyte ratio, GLR: Granulocyte-lymphocyte ratio, PLR: Platelet-lymphocyte ratio, TB-Total Bilirubin, AST-Aspartate aminotransferase, ALT-Alanine aminotransferase, ALP-Alkaline phosphatase

### A comparative analysis of HLA-G levels, lymphocyte-monocyte ratio (LMR), granulocyte-lymphocyte ratio (GLR), and platelet-lymphocyte ratio (PLR) between malaria-infected and uninfected pregnant women

**Fig 1:**
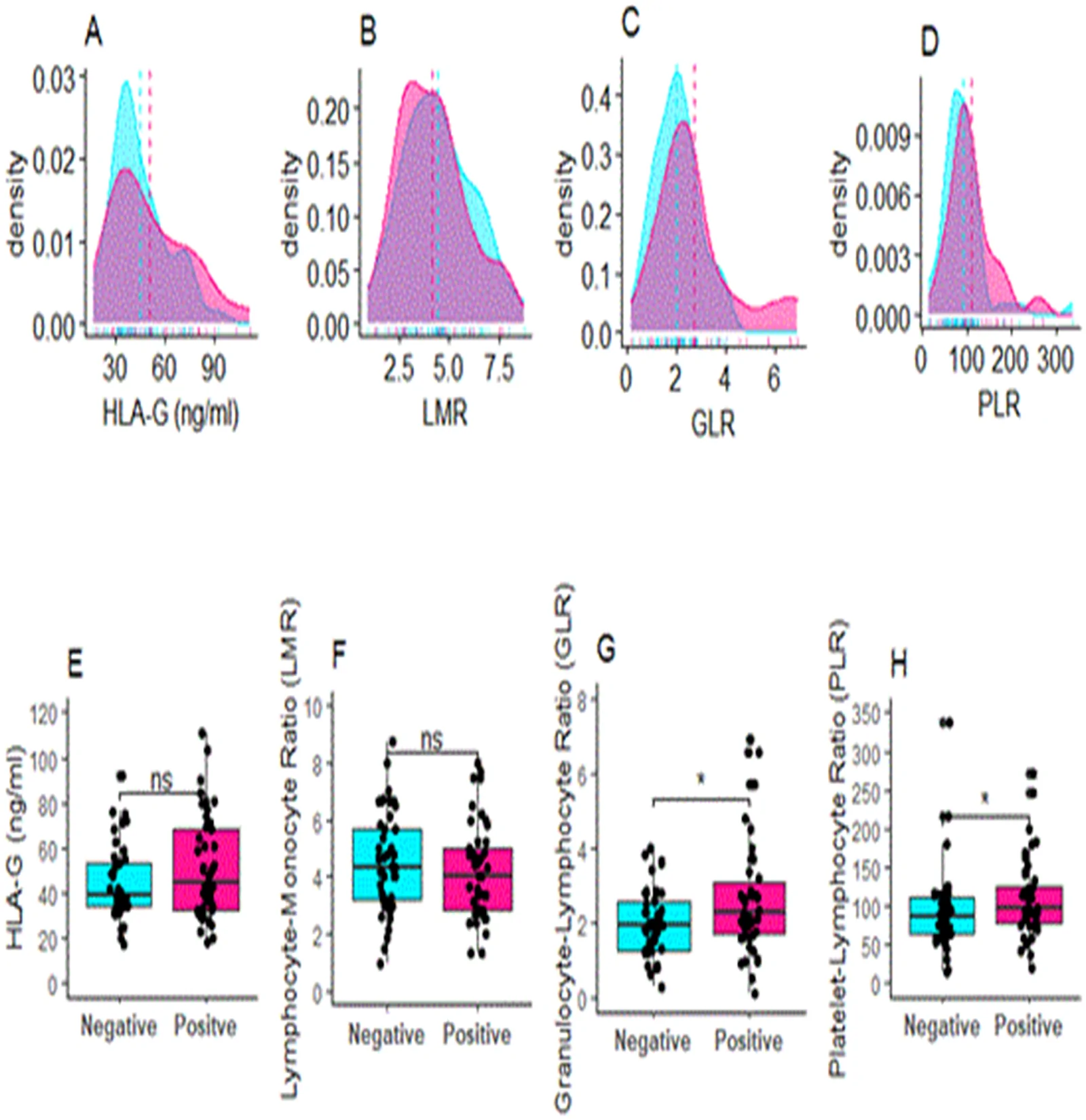
Density (A-D) and Box and Whiskers Plot (E-H) Depicting Comparison of Levels of HLA-G, Lymphocyte-Monocyte Ratio (LMR), Granulocyte-Lymphocyte Ratio (GLR) and Platelet-Lymphocyte Ratio (PLR) Between pregnant women with and without malaria infection.Note: *p*-values computed by Mann-Whitney U-test, ns: not significant, *: *p* < 0.05, **: *p* < 0.01, ***: *p* < 0.001, ****: *p* < 0.0001.

This study observed that GLR (G) and PLR (H) levels were significantly higher in participants with *Pf* infections compared to participants without *Pf* infection (*p* < 0.05). HLA-G levels were also slightly higher in *Pf*-positive subjects than in *Pf*-negative subjects, however not statistically significant (*p* > 0.05). Besides, LMR levels were slightly lower in *Pf*-positive subjects than in *Pf*-negative subjects, although not statistically significant (*p* > 0.05) (Fig 1).

### HLA-G levels and hematobiochemical parameters associated with miscarriage among malaria-infected and uninfected pregnant women

We found that women with a history of miscarriage had considerably higher HLA-G levels than women with no history of miscarriage. Meanwhile, GLR levels were significantly higher among the malaria-infected than the negative group within the non-miscarriage group (2.52ng/ml vs. 1.87ng/ml, p = 0.039). (Table 4).

**Table 4:** HLA-G levels and hematobiochemical parameters associated with miscarriage among malaria-infected and uninfected pregnant women.

Variables	No Miscarriage (n = 61)	Miscarriage (n = 31)	*p*-value
**HLA-G (ng/mL)**	35.44 (31.84-42.72)	50.14 (36.30-71.00)	**0.001**
*Pf Negative*	39.15 (32.25-44.89)	48.14 (34.36-60.91)	0.223
*Pf Positive*	32.39 (26.42-44.65)	55.57 (38.23-79.10)	**0.001**
*p-value*	0.078	0.215	
**LMR**	4.28 ± 1.74	4.40 ± 1.66	0.756
*Pf Negative*	4.29 ± 1.76	4.76 ± 1.73	0.390
*Pf Positive*	4.28 ± 1.74	3.97 ± 1.51	0.571
*p-value*	0.972	0.193	
**GLR**	2.21 ± 1.23	2.61 ± 1.50	0.180
*Pf Negative*	1.87 ± 0.86	2.22 ± 0.89	0.205
*Pf Positive*	2.52 ± 1.44	3.09 ± 1.93	0.277
*p-value*	**0.039**	0.107	
**PLR**	97.73 ± 51.58	107.03 ± 52.16	0.403
*Pf Negative*	85.13 ± 56.31	104.37 ± 41.90	0.228
*Pf Positive*	108.57 ± 44.90	110.25 ± 64.01	0.919
*p-value*	0.076	0.761	
**TB (μmol/L)**	8.57 (5.39-16.58)	11.61 (7.82-14.49)	0.480
*Pf Negative*	7.62 (4.64-15.98)	8.81 (6.12-14.16)	0.882
*Pf Positive*	9.10 (5.48-18.65)	12.57 (9.84-20.82)	0.328
*p-value*	0.394	0.100	
**AST (U/L)**	25.40 (15.76-41.15)	30.50 (18.88-45.94)	0.336
*Pf Negative*	30.45 (19.10-44.50)	39.75 (21.56-51.61)	0.446
*Pf Positive*	19.45 (7.81-33.76)	20.13 (15.95-34.16)	0.616
*p-value*	0.053	0.084	
**ALT (U/L)**	11.37 (5.90-18.24)	14.43 (10.12-24.47)	0.054
*Pf Negative*	12.35 (7.07-21.18)	17.48 (10.43-27.08)	0.199
*Pf Positive*	10.17 (5.34-15.54)	11.63 (9.71-15.99)	0.174
*p-value*	0.069	0.071	
**ALP (U/L)**	207.48 (146.35-282.06)	201.05 (150.25-296.33)	0.928
*Pf Negative*	219.45 (183.80-294.03)	215.86 (160.07-300.75)	0.625
*Pf Positive*	183.01 (118.76-279.57)	193.05 (135.75-275.27)	0.591
*p-value*	0.057	0.444	

**Note**: Comparison of parametric continuous variables (represented as means and standard deviations) between subjects with and without miscarriage were computed by Independent Sample T-Test whilst comparison of non-parametric continuous variables (represented as median and interquartile ranges) between subjects with and without miscarriage were computed by Mann-Whitney U-Test; Similarly, comparison of parametric continuous and non-parametric continuous variables between *Pf* negative and *Pf* positive subjects were computed by Independent Sample T-Test and Mann-Whitney U-Test respectively; *p*-values < 0.05 and bolded means statistically significant

**Abbreviations:** HLA-G- Human Leukocyte Antigen-G, LMR: Lymphocyte-monocyte ratio, GLR: Granulocyte-lymphocyte ratio, PLR: Platelet-lymphocyte ratio, TB-Total Bilirubin, AST-Aspartate aminotransferase, ALT-Alanine aminotransferase, ALP-Alkaline phosphatase

## Discussion

The immune-suppressive properties of HLA-G and its interaction with the fetal-maternal interface contribute to the susceptibility of pregnant women to *Pf* infection and pregnancy loss [18]. Changes in hematological parameters can exacerbate the severity of malaria infections [19]. Furthermore, malaria infection can cause liver damage in hepatocytes, posing a threat to both the mother and fetus. However, studies have reported inconsistent results regarding HLA-G levels among pregnant women with *Pf* infections. Therefore, this study evaluated the levels of HLA-G and the haematobiochemical profiles of pregnant women in Ghana, both infected and uninfected with *Pf.*

The significant association between non-use of IPTp-SP and *Pf* positivity is consistent with established evidence that sulfadoxine-pyrimethamine-based chemoprophylaxis reduces maternal and placental malaria infection, miscarriage risk, and low birth weight in sub-Saharan Africa [20,21]. Updated WHO 2022 guidelines recommend IPTp-SP for all pregnant women regardless of gravidity [22], yet uptake in Ghana remains below the 80% national target [23]. The similar ITN usage across groups suggests that bed net coverage alone was insufficient to modify *Pf* risk in this cohort, underscoring the primacy of pharmacological prophylaxis.

Our results did not show a statistically significant difference in miscarriage rates between Pf-infected (30.4%) and *Pf-*uninfected (37.0%) subjects (p = 0.508). Studies have reported inconsistent findings regarding miscarriage and *P. falciparum* infections. A study by [24] found substantially different results in a large population-based study of 17,613 pregnant women from the Thai-Burmese border, reporting that first-trimester malaria significantly increased miscarriage risk (adjusted odds ratio 2.70 for asymptomatic and 3.99 for symptomatic Pf infection). However, [25] examined first-trimester *Pf* infection across Kenya, Zambia, and the Democratic Republic of the Congo in 282 women and found no significant association with spontaneous abortion, aligning with the reported finding (adjusted prevalence differences near zero across all sites). These conflicting results suggest that the relationship between first-trimester *Pf infection* and miscarriage may vary by geographic setting, transmission intensity, population characteristics and sample size. The heterogeneity in findings warrants further investigation to clarify when and under what conditions *Pf infection* increases miscarriage risk.The persistence of significantly elevated ALP in *Pf*-infected women after adjustment for age points to a pathological increment above the expected physiological gestational rise. During normal pregnancy, ALP increases progressively to two to four times non-pregnant reference values due to placental isoenzyme production by syncytiotrophoblasts [26]. Thus, an ALP elevation that survives covariate adjustment in malaria-infected women suggests an additional malaria-related contribution. This is consistent with malaria-associated liver dysfunction (MALID), in which intrahepatic cholestasis secondary to reticuloendothelial blockage, parasitised erythrocyte sinusoidal sequestration, and haemozoin-driven hepatocyte membrane injury collectively elevate cholestatic markers such as ALP [27,28]. In contrast, the attenuation of the ALT association after age adjustment aligns with the physiological depression of transaminase levels during pregnancy attributable to haemodilution and hormonal influences, which limits the discriminatory sensitivity of ALT in this population [29]. These findings suggest that ALP is a more robust marker of malaria-related hepatic involvement in pregnant women than ALT, and that routine monitoring of ALP trends during antenatal care may assist in the early detection of hepatic complications in *Pf*-infected women.

The significant elevation of GLR and PLR in *Pf*-infected women (Fig 1) and the unadjusted association between increasing GLR and *Pf* infection are consistent with the pattern of granulocyte activation and thrombocytopenia characteristic of falciparum malaria., Granulocyte recruitment is driven by parasite-antigen-induced innate immune activation whilst relative lymphopenia results from apoptosis and lymphocyte redistribution to secondary lymphoid organs during acute parasitaemia [30,31]. Thrombocytopenia, the most consistent haematological hallmark of *Pf* infection, reduces absolute platelet counts through splenic sequestration and complement-mediated platelet destruction, thereby elevating PLR [32]. These findings are corroborated by a recent Ghanaian cross-sectional study that demonstrated significantly elevated NLR and PLR in *Pf*-infected individuals compared with controls [30], and a 2025 study confirming elevated FBC-derived inflammatory indices in malaria patients [31]. The loss of significance in adjusted models likely reflects the small sample size and multicollinearity among haematological covariates rather than an absence of biological effect. LMR was non-significantly lower in infected women, consistent with attenuated immune dysregulation expected in semi-immune multigravid populations [33]. Collectively, GLR and PLR represent low-cost, routinely available inflammatory indicators with practical utility in resource-limited Ghanaian antenatal settings.

HLA-G levels were slightly but non-significantly higher in *Pf*-positive women. Although statistical significance was not reached, likely due to limited sample size, the directional finding is biologically coherent. HLA-G is a non-classical MHC class I molecule expressed on trophoblast cells that mediates maternal-foetal immune tolerance by suppressing NK cell cytotoxicity and inhibiting T-cell responses [34]. Plasmodium may exploit this tolerogenic pathway to evade host immunity. Courtin et al. demonstrated that elevated soluble HLA-G in maternal blood was associated with increased infant susceptibility to malaria, and d'Almeida et al. confirmed that mothers with placental malaria were more likely to give birth to children with high sHLA-G — consistent with HLA-G-mediated immunosuppression creating a permissive environment for parasite survival [35,36]. These findings motivate adequately powered prospective studies with concurrent placental HLA-G measurement to clarify this relationship.

Women with a history of miscarriage had considerably higher circulating HLA-G levels than those without. This finding is consistent with the immunological literature describing elevated soluble HLA-G as a systemic response to recurrent trophoblastic stress or prior placental injury [8]. Although reduced trophoblastic HLA-G expression is associated with recurrent pregnancy loss through failure to suppress NK cell-mediated cytolysis [37], elevated peripheral sHLA-G may represent a compensatory upregulation following repeated episodes of placental damage. Madduru et al. demonstrated that reduced sHLA-G5 isoforms combined with an elevated TNF-α:IL-4 ratio characterise women with recurrent pregnancy loss, suggesting that immune dysregulation rather than HLA-G level alone determines pregnancy outcome [38]. Within the non-miscarriage subgroup, GLR remained significantly elevated in malaria-infected compared with uninfected women, confirming that malarial granulocyte activation is a consistent haematological feature of *Pf* infection independent of pregnancy loss history.

The simultaneous assessment of HLA-G, FBC-derived inflammatory indices, and hepatic enzymes within a single case-control framework is the first to be conducted within the Ghanaian population. Nonetheless, we acknowledge a few limitations of the study. The relatively small sample size constrains statistical power, particularly for subgroup analyses, leading to a lack of generalizability. Also, the self-reporting of miscarriage status, IPT-SP use and by participants could result in recall bias. The cross-sectional design precludes causal inference, and HLA-G measured in peripheral rather than placental blood may not fully reflect local immunological dynamics at the maternal-foetal interface. Finally, the use of purposive sampling could introduce systematic bias. Further longitudinal studies with a large sample size are required to confirm the findings of this study.

## Conclusion

*Pf* infection in pregnant Ghanaian women was associated with elevated ALP, GLR, and PLR, indicating hepatic dysfunction and systemic inflammation. The non-use of IPTp-SP among pregnant women was significantly associated with active *Pf* infection, highlighting the importance of chemoprophylaxis. Increased sHLA-G levels in women with miscarriage suggest an immunological link to pregnancy loss. Further prospective investigation of sHLA-G as a biomarker of adverse pregnancy outcome in malaria-endemic settings with a larger sample size is required.

## Supporting information

S1 Appendix HLAG manuscript data. (XLSX)

## References

[1] WHO. World malaria report. World Health Organization. 2023. https://www.who.int/news-room/fact-sheets/detail/malaria

[2] Uneke CJ. Impact of placental Plasmodium falciparum malaria on pregnancy and perinatal outcome in sub-Saharan Africa: I: introduction to placental malaria. Yale J Biol Med. 2007;80(2):39–50. 18160989 PMC2140183

[3] Obeagu E, Obeagu G. Maternal malaria: implications for fetal health. Elite Journal of Nursing and Health Science. 2024;2(6):69–89.

[4] Kapisi J, Kakuru A, Jagannathan P, Muhindo MK, Natureeba P, Awori P, et al. Relationships between infection with Plasmodium falciparum during pregnancy, measures of placental malaria, and adverse birth outcomes. Malar J. 2017;16(1):400. doi: 10.1186/s12936-017-2040-4 28982374 PMC5629777

[5] Arnaiz-Villena A, Suarez-Trujillo F, Juarez I, Rodríguez-Sainz C, Palacio-Gruber J, Vaquero-Yuste C, et al. Evolution and molecular interactions of major histocompatibility complex (MHC)-G, -E and -F genes. Cell Mol Life Sci. 2022;79(8):464. doi: 10.1007/s00018-022-04491-z 35925520 PMC9352621

[6] Ferreira LMR, Meissner TB, Tilburgs T, Strominger JL. HLA-G: At the Interface of Maternal-Fetal Tolerance. Trends Immunol. 2017;38(4):272–86. doi: 10.1016/j.it.2017.01.009 28279591

[7] Amiot L, Vu N, Samson M. Immunomodulatory properties of HLA-G in infectious diseases. J Immunol Res. 2014;2014:298569. doi: 10.1155/2014/298569 24839609 PMC4009271

[8] Barbaro G, Inversetti A, Cristodoro M, Ticconi C, Scambia G, Di Simone N. HLA-G and Recurrent Pregnancy Loss. Int J Mol Sci. 2023;24(3):2557. doi: 10.3390/ijms24032557 36768880 PMC9917226

[9] Koc A, Kirbiyik O, Kutbay YB, Ozyilmaz B, Ozdemir TR, Kaya OO, et al. Fetal HLA-G alleles and their effect on miscarriage. Adv Clin Exp Med. 2018;27(9):1233–7. doi: 10.17219/acem/69692 29809322

[10] Adamu J, Jigam A. Effects of Malaria Infection on some Haematological and Biochemical Parameters in the General Population and Pregnant Malaria Patients Attending Two District Hospitals in Niger State, Nigeria. Glob J Infect Dis Clin Res. 2019;:001–5. doi: 10.17352/2455-5363.000021

[11] Maina RN, Walsh D, Gaddy C, Hongo G, Waitumbi J, Otieno L, et al. Impact of Plasmodium falciparum infection on haematological parameters in children living in Western Kenya. Malar J. 2010;9 Suppl 3(Suppl 3):S4. doi: 10.1186/1475-2875-9-S3-S4 21144084 PMC3002140

[12] Okorie H, Obeagu E, Eze E, Jeremiah Z. Assessment of some haematological parameters in malaria infected pregnant women in Imo state Nigeria. Int J Curr Res Biol Med. 2018;3(9):1–4.

[13] Anoosha G. Liver function abnormalities and its prognostic significance in malaria. India: Rajiv Gandhi University of Health Sciences. 2016.

[14] Kumi E, Daymond A. Farmers’ Perceptions of the Effectiveness of the Cocoa Disease and Pest Control Programme (CODAPEC) in Ghana and Its Effects on Poverty Reduction. AJEA. 2015;7(5):257–74. doi: 10.9734/ajea/2015/16388

[15] Service GS. Sefwi bibiani-anhwiaso- bekwai district 2022 [.

[16] Ba EH, Baird JK, Barnwell J, Bell D, Carter J, Dhorda M, et al. Microscopy for the detection, identification and quantification of malaria parasites on stained thick and thin blood films in research settings: procedure: methods manual. 2015.

[17] Fiseha M, Mohammed M, Ebrahim E, Demsiss W, Tarekegn M, Angelo A, et al. Common hematological parameters reference intervals for apparently healthy pregnant and non-pregnant women of South Wollo Zone, Amhara Regional State, Northeast Ethiopia. PLoS One. 2022;17(7):e0270685. doi: 10.1371/journal.pone.0270685 35839211 PMC9286272

[18] Sadissou I, d’Almeida T, Cottrell G, Luty A, Krawice-Radanne I, Massougbodji A, et al. High plasma levels of HLA-G are associated with low birth weight and with an increased risk of malaria in infancy. Malar J. 2014;13:312. doi: 10.1186/1475-2875-13-312 25115633 PMC4248443

[19] Das S, Rajkumari N, Chinnakali P. A comparative study assessing the effect of haematological and biochemical parameters on the pathogenesis of malaria. J Parasit Dis. 2019;43(4):633–7. doi: 10.1007/s12639-019-01142-2 31749535 PMC6841811

[20] Radeva-Petrova D, Kayentao K, ter Kuile FO, Sinclair D, Garner P. Drugs for preventing malaria in pregnant women in endemic areas: any drug regimen versus placebo or no treatment. Cochrane Database Syst Rev. 2014;2014(10):CD000169. doi: 10.1002/14651858.CD000169.pub3 25300703 PMC4498495

[21] Menéndez C, Bardají A, Sigauque B, Sanz S, Aponte JJ, Mabunda S, et al. Malaria prevention with IPTp during pregnancy reduces neonatal mortality. PLoS One. 2010;5(2):e9438. doi: 10.1371/journal.pone.0009438 20195472 PMC2829080

[22] WHO. WHO guidelines for malaria. World Health Organization. 2022.

[23] Ampofo GD, Ahiakpa AK, Osarfo J. Interventions for malaria prevention in pregnancy; factors influencing uptake and their effect on pregnancy outcomes among post-natal women in a tertiary facility in the Volta Region of Ghana. SAGE Open Med. 2023;11:20503121231199653. doi: 10.1177/20503121231199653 37719169 PMC10503280

[24] McGready R, Lee SJ, Wiladphaingern J, Ashley EA, Rijken MJ, Boel M, et al. Adverse effects of falciparum and vivax malaria and the safety of antimalarial treatment in early pregnancy: a population-based study. Lancet Infect Dis. 2012;12(5):388–96. doi: 10.1016/S1473-3099(11)70339-5 22169409 PMC3346948

[25] Leuba SI, Westreich D, Bose CL, Olshan AF, Taylor SM, Tshefu A, et al. Effects on maternal and pregnancy outcomes of first-trimester malaria infection among nulliparous women from Kenya, Zambia, and the Democratic Republic of the Congo. PLoS One. 2024;19(12):e0310339. doi: 10.1371/journal.pone.0310339 39705264 PMC11661578

[26] Ferro B, Marques I, Paixão J, Almeida M do C. Incidental Finding of Extreme Elevation of Serum Alkaline Phosphatase in Pregnancy. Cureus. 2021;13(8):e17211. doi: 10.7759/cureus.17211 34540438 PMC8442801

[27] Prenen F, Van den Steen PE. Malaria-associated liver dysfunction: a forgotten challenge. Trends Parasitol. 2025;41(7):547–59. doi: 10.1016/j.pt.2025.05.010 40480922

[28] Megabiaw F, Eshetu T, Kassahun Z, Aemero M. Liver enzymes and lipid profile of malaria patients before and after antimalarial drug treatment at Dembia Primary Hospital and Teda Health Center, Northwest, Ethiopia. Research and Reports in Tropical Medicine. 2022;:11–23. 35370434 10.2147/RRTM.S351268 PMC8974243

[29] Dajti E, Bruni A, Barbara G, Azzaroli F. Diagnostic Approach to Elevated Liver Function Tests during Pregnancy: A Pragmatic Narrative Review. J Pers Med. 2023;13(9):1388. doi: 10.3390/jpm13091388 37763154 PMC10532949

[30] Bohli JK, Ansah PB, Kwamena TE, Allotey E, Duneeh RV, Yeboah EB, et al. Variations in haematological and inflammatory biomarkers and their association with Plasmodium falciparum malaria: a cross-sectional comparative study at a clinic in Ghana. Malar J. 2025;24(1):220. doi: 10.1186/s12936-025-05474-8 40629396 PMC12235773

[31] Boachie J, Ahiable D, Ajabuin LA, Amissah R, Asmah-Brown A, Apalebilah S, et al. Diagnostic value of full blood count derived systemic inflammatory biomarkers in malaria infection. Pract Lab Med. 2025;46:e00494. doi: 10.1016/j.plabm.2025.e00494 40756261 PMC12318341

[32] Lacerda MVG, Mourão MPG, Coelho HCC, Santos JB. Thrombocytopenia in malaria: who cares?. Memórias do Instituto Oswaldo Cruz. 2011;106(suppl 1):52–63. 21881757 10.1590/s0074-02762011000900007

[33] Antwi-Baffour S, Kyeremeh R, Buabeng D, Adjei JK, Aryeh C, Kpentey G, et al. Correlation of malaria parasitaemia with peripheral blood monocyte to lymphocyte ratio as indicator of susceptibility to severe malaria in Ghanaian children. Malar J. 2018;17(1):419. doi: 10.1186/s12936-018-2569-x 30419923 PMC6233557

[34] Carosella ED, Rouas-Freiss N, Tronik-Le Roux D, Moreau P, LeMaoult J. HLA-G: An Immune Checkpoint Molecule. Adv Immunol. 2015;127:33–144. doi: 10.1016/bs.ai.2015.04.001 26073983

[35] Sadissou I, d’Almeida T, Cottrell G, Luty A, Krawice-Radanne I, Massougbodji A, et al. High plasma levels of HLA-G are associated with low birth weight and with an increased risk of malaria in infancy. Malar J. 2014;13:312. doi: 10.1186/1475-2875-13-312 25115633 PMC4248443

[36] d’Almeida TC, Sadissou I, Sagbohan M, Milet J, Avokpaho E, Gineau L, et al. High level of soluble human leukocyte antigen (HLA)-G at beginning of pregnancy as predictor of risk of malaria during infancy. Sci Rep. 2019;9(1):9160. doi: 10.1038/s41598-019-45688-w 31235762 PMC6591392

[37] Mosaferi E, Alizadeh Gharamaleki N, Farzadi L, Majidi J, Babaloo Z, Kazemi T, et al. The Study of HLA-G Gene and Protein Expression in Patients with Recurrent Miscarriage. Adv Pharm Bull. 2019;9(1):70–5. doi: 10.15171/apb.2019.009 31011560 PMC6468217

[38] Madduru D, Dirisipam K, Goli M, Ramana Devi V, Jahan P. Association of reduced maternal sHLA-G5 isoform levels and elevated TNF-α/IL-4 cytokine ratio with Recurrent Pregnancy Loss: A study on South Indian women. Scand J Immunol. 2021;94(4):e13095. doi: 10.1111/sji.13095 34780078

